# Pore-Scale Imaging to Quantify the Evolution and Reduction in Trapped CO_2_ due to Ostwald Ripening

**DOI:** 10.1021/acs.est.5c06424

**Published:** 2025-12-01

**Authors:** Rukuan Chai, Sajjad Foroughi, Sepideh Goodarzi, Anindityo Patmonoaji, Foo Yoong Yow, Branko Bijeljic, Martin J. Blunt

**Affiliations:** † Department of Earth Science and Engineering, 4615 Imperial College London, London SW7 2AZ, U.K.; ‡ Petroliam Nasional Berhad, PETRONAS, Kajang, Selangor 43000, Malaysia

**Keywords:** geological CO_2_ storage, pore-scale imaging, reservoir sandstone, Ostwald ripening, ganglia rearrangement, trapped saturation reduction

## Abstract

Geological carbon storage is a key strategy for mitigating climate change, but the long-term stability of trapped CO_2_ remains uncertain. Transport of dissolved CO_2_ in the aqueous phase can cause the rearrangement of capillary-trapped CO_2_ in the pore space, which is called Ostwald ripening. Using high-resolution three-dimensional X-ray imaging, we visualized the in situ evolution of CO_2_ ganglia in reservoir sandstone during storage and quantified its impact on trapped CO_2_ saturation. Pore-scale imaging showed the concurrent shrinkage and growth of CO_2_ ganglia, reduced morphological complexity, and enhanced connectivity, resulting from Ostwald ripening. Ganglia exhibited a size-dependent response: small ganglia dissolved and disappeared, intermediate ones shrank or grew, and large ganglia stabilized with occasional fragmentation. After waiting for 58 h with no flow, originally residual CO_2_ reconnected, and subsequent brine injection led to a decrease in saturation from 22.8% to 15.6%, consistent with previous estimates based on pore-scale modeling. This work suggests that measurements that ignore the effect of Ostwald ripening overestimate the residual saturation by a factor of approximately a third.

## Graphical abstract

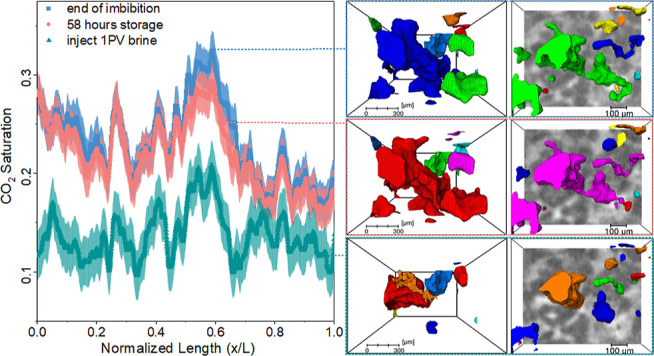

## 1 Introduction

Geological CO_2_ storage is a vital technology for mitigating greenhouse gas emissions and achieving global net-zero targets.^1–3^ Successful implementation of CO_2_ sequestration and long-term storage stability critically depends on a thorough understanding of CO_2_ flow and trapping mechanisms. In recent years, significant progress has been made in characterizing CO_2_ multiphase flow dynamics,^1,4^ as well as in elucidating the impacts of reservoir heterogeneity^5^ and geochemical reactions.^6,7^ However, the dynamics of stored CO_2_ following injection, particularly during trapping, remain poorly understood. This limitation largely stems from the fact that much of the current knowledge of multiphase flow in porous media is derived from experience with hydrocarbon recovery,^8^ where it is assumed that oil and water are completely immiscible, and hence once oil is surrounded by brine in the pore space, it remains trapped.^9,10^ This capillary trapping process has also been observed for CO_2_;^11,12^ however, in early studies, the rearrangement and reconnection of trapped ganglia mediated by transport of dissolved CO_2_ in the aqueous phase, called Ostwald ripening, was ignored.

Multiple experiments^13–19^ and numerical modeling^20–23^ have recently investigated Ostwald ripening, demonstrating that it can drive substantial mass redistribution in porous media. X-ray computed tomography (CT) imaging studies by Garing et al.,^13^ Zhang et al.,^15^ Goodarzi et al.,^14,16^ and Boon et al.,^17^ along with microfluidic observations by Xu et al.,^24^ have revealed that gas ganglia can enlarge and become more connected. Pore-scale modeling by Singh et al.^21,22^ further showed that larger ganglia become increasingly ramified at the expense of smaller, spherical ones. Similar phenomena, including shrinkage and growth of trapped bubbles or foams, were observed in micromodel experiments by Huang et al.,^25^ Xu et al.,^18^ Wang et al.,^26^ and Feng et al.^19^ Joewondo et al.^27^ combined microfluidic experiments with pore-network modeling to show that larger bubbles exhibited continuous growth, while smaller bubbles initially grew but eventually dissolved. Adebimpe et al.^23^ used a percolation-based pore-network model to predict the impact of Ostwald ripening on residual gas trapping. Their work assumed that local capillary equilibrium is achieved at the scale of a few pores, which typically takes on the order of days to weeks for most gases.^28^ Once equilibrium is established, further displacement of the initially trapped gas becomes possible, ultimately leading to lower residual saturation. Although previous studies suggest that Ostwald ripening rearranges trapped ganglia and may reduce gas trapping, direct experimental confirmation is lacking. Moreover, existing investigations have primarily focused on ganglia size evolution, typically reporting only statistical trends, while the detailed dynamics of ganglion transformation remains poorly understood. In addition, most prior experiments were conducted in micromodels or homogeneous quarry sandstones, which do not capture the geological complexity of actual or planned CO_2_ storage reservoirs.

This study visualizes the in situ Ostwald ripening of CO_2_ ganglia in reservoir sandstone and quantitatively evaluates its impact on residual saturation using high-resolution three-dimensional X-ray imaging during controlled displacement experiments. Uniquely, we tracked the individual evolution of ganglia over time, providing a detailed pore-scale characterization of the ganglion dynamics during Ostwald ripening. Our observations enable refinement of multiphase flow properties used in field-scale modeling, ensuring that the long-term impacts of Ostwald ripening are properly incorporated into CO_2_ storage predictions.

## 2 Materials and Methods

### 2.1 Materials

A cylindrical sandstone sample, obtained from a depleted gas reservoir that is a proposed site for CO_2_ storage, was used for the experiment. The sample measured 6.0 ± 0.1 mm in diameter and 23.7 ± 0.1 mm in length with a porosity of 25.4% and an absolute permeability of 200 ± 10 mD. Its mineralogical composition consisted of 73.6% quartz, 16.6% plagioclase, 2.8% calcite, 0.2% siderite, and 6.8% clay. To enhance X-ray attenuation contrast among the brine, CO_2_, and rock phases during micro-CT scanning and facilitate subsequent image processing and analysis, a 25 wt % potassium iodide (KI)-doped brine^7,29,30^ was used in all the experiments. Although the addition of KI may influence physicochemical properties, including CO_2_ solubility and mass transfer rates, the effect on the patterns of Ostwald ripening of CO_2_ ganglia, the primary focus of this work, is unlikely to be significant.

### 2.2 Methods

Figure 1 illustrates the experimental apparatus. The system comprised five pumps—for brine equilibration and injection, CO_2_ injection, confining pressure, and back pressure regulation—along with a high-pressure reactor, a core holder, and a pressure transducer, all interconnected via polyether–ether–ketone (PEEK) tubing. The core holder’s temperature was regulated by a proportional-integral-derivative (PID) controller that drove a heater jacket with thermocouple feedback, while the temperatures of the pumps and tubing were maintained using a recirculating water bath. X-ray micro-CT images were acquired by using a Zeiss Xradia 510 Versa X-ray microscope.

**Figure 1:**
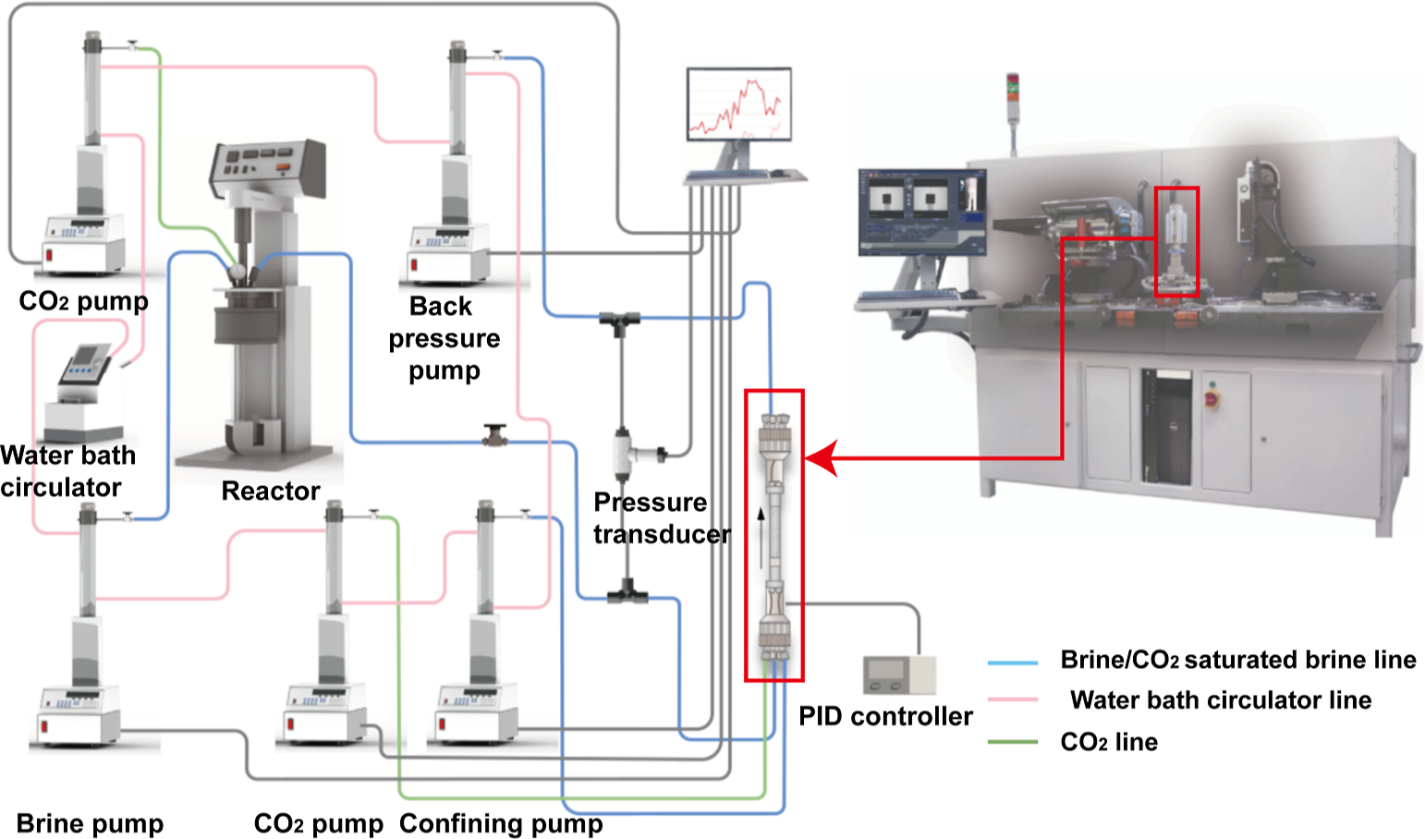
Schematic diagram of the experimental apparatus.

Residual sandstone, remaining after drilling the cylindrical sample, was fragmented into small pieces and placed in a high-pressure reactor. 600 mL of 25 wt % KI-doped brine was added, and the reactor was sealed, heated to 50 °C, and pressurized with CO_2_ to 8 MPa for 2 weeks to achieve thermodynamic equilibrium between the brine, CO_2_, and rock phases, yielding CO_2_-saturated brine and brine-saturated CO_2_. The cylindrical sample was dried in an oven at 60 °C for 2 days and then enclosed in a Viton sleeve and mounted into a core holder. The assembled core holder was then installed vertically in the CT scanner and connected to the fluid delivery and receiving system, the pressure transducer, and other experimental components. For the small sample used, gravitational effects are likely to be negligible, as confirmed by detailed calculations in Text S1 of the Supporting Information. A confining pressure of 2 MPa was applied to ensure proper sealing and eliminate the bypass flow between the sample and sleeve. CO_2_ was injected for 30 min to flush residual fluids and fines, followed by 12 h of vacuuming to evacuate remaining gases. A dry scan of the sample was first performed. Brine was injected at a flow rate of 0.20 mL/min while the system was gradually brought to reservoir conditions (8 MPa and 50 °C), followed by a scan after the system pressure had stabilized for over 2 h. Subsequently, CO_2_ was injected at the same flow rate until the pressure restabilized and remained steady for at least 2 h. Following this drainage step, brine was reinjected at 0.20 mL/min, and a scan was acquired after pressure stabilization for 2 h. To investigate Ostwald ripening, the system was then held under static conditions (constant pressure, no flow) for 58 h to allow trapped CO_2_ ganglia to equilibrate via diffusion-driven processes, followed by another scan. The waiting period was based on estimates of the time for Ostwald ripening to have a significant effect throughout the sample, as detailed in Text S2 of the Supporting Information. While changes in pressure as the system reaches equilibrium could change the balance of dissolved and free-phase CO_2_, this effect is likely to be small. Finally, 1 pore volume of brine was injected to displace any mobilized CO_2_ resulting from Ostwald ripening, and a final scan was performed. For each stage, a scan of the whole sample (voxel size: 4.5 μm) was conducted to quantify CO_2_ saturation, accompanied by a higher-resolution, zoomed-in image (voxel size: 1.82 μm) focusing on the central region of the sample to characterize the size, morphology, and connectivity of trapped ganglia. The capillary number was 6.6 × 10^–8^, based on the definition $Ca=\frac{\mu q}{\sigma }$ ,^7,8^ where μ is the viscosity of the CO_2_, *q* is the Darcy velocity, and σ is the interfacial tension between the two phases; this value remained in the capillary-dominated regime, similar to reservoir conditions.

Imaging was conducted at 90 keV X-ray energy and 8 W power. Each scan consisted of 3001 projections with a 2 s exposure time and 1× binning. Images were reconstructed using Zeiss Reconstructor software with corrections applied for center shift and beam hardening. Following reconstruction, the image processing and analysis were performed using commercial image analysis software, Avizo. Specifically, images were denoised using a nonlocal means filter, normalized to grayscale, and spatially registered to the dry scan using Lanczos resampling. Phase segmentation was conducted by combining differential imaging with a marker-based watershed method.^7^ In this process, the multiphase image was subtracted from the fully brine-saturated image to highlight CO_2_, while the subtraction of the dry scan from the multiphase image enhanced the brine phase. Both CO_2_ and brine were then segmented using the watershed algorithm, and the remaining voxels were classified as sandstone minerals. Finally, quantitative analysis of CO_2_ saturation and CO_2_ ganglia properties, including volume, surface area, and Euler characteristics, was performed.

Additionally, we tracked the temporal evolution of individual CO_2_ ganglion volumes to characterize Ostwald ripening at the pore scale. First, all discrete CO_2_ regions were identified in each three-dimensional image using connected-component labeling with each ganglion assigned a unique identifier. Ganglia smaller than 27 voxels were excluded from further analysis since they may be segmentation artifacts. To track ganglia over time, spatial correspondence between consecutive images was established by evaluating voxel-wise overlaps. A ganglion was classified as complete dissolution if it had no overlap in the subsequent image. If a ganglion mapped to a single ganglion in the next image and the mapping was mutual, it was considered stable if its volume changed by less than 1% or was categorized as growth or shrinkage depending on whether the volume increased or decreased. If a ganglion split into multiple parts, the event was classified as fragmentation; if multiple ganglia merged into one, it was classified as coalescence. Ganglia that newly appeared without any overlap in the previous image were defined as nucleation growth.

## 3 Results and Discussion

### 3.1 Reduction in Trapped CO_2_ Saturation

The raw and segmented images at different stages, along with the CO_2_ saturation profile and porosity distribution, are shown in Figure 2. As illustrated in Figure 2a–d, after several hundred pore volumes of brine flushing, CO_2_, as a nonwetting phase in this reservoir sandstone,^7^ was immobilized as discrete ganglia within relatively large pores. The average trapped CO_2_ saturation was 22.8%, as depicted in Figure 2e. This residual trapping results from capillary forces that induce snap-off at restrictions in the pore space, called throats, disconnecting and immobilizing the nonwetting CO_2_ in pore bodies^11,31,32^ and preventing its further mobilization under typical reservoir conditions. Furthermore, CO_2_ saturation qualitatively correlated with local porosity, with higher-porosity regions generally retaining greater amounts of CO_2_ (Figures 2e and 2f). This correlation stems from capillary pressure scaling inversely with throat radius:^8^ larger throats enable easier CO_2_ invasion into associated larger pores during drainage, followed by preferential residual trapping during subsequent imbibition.^33^

**Figure 2:**
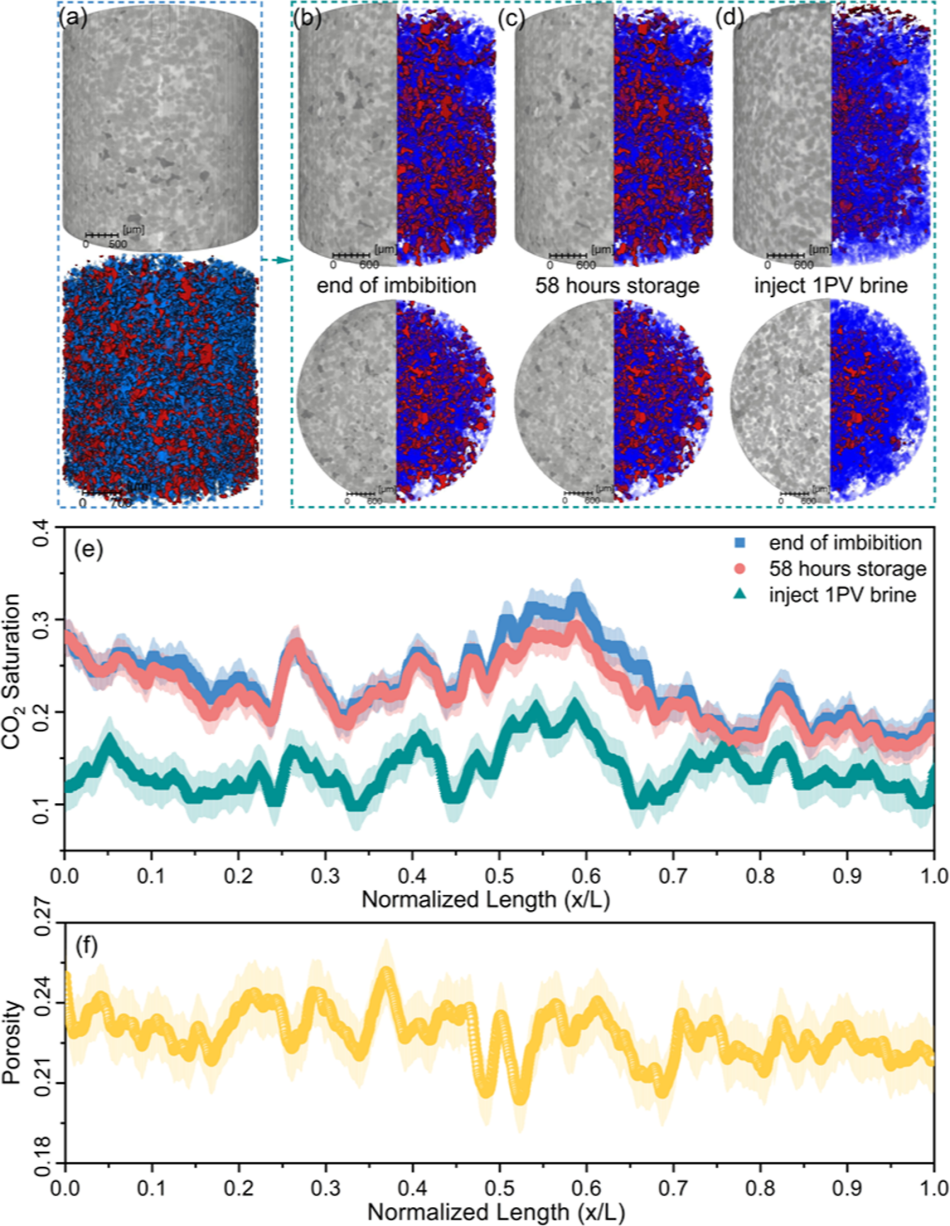
Effect of Ostwald ripening on trapped CO_2_ saturation. (a) Schematic illustration of the procedure used to generate three-dimensional visualizations and cross-sectional images of both raw (bright: brine; gray: sandstone; dark: CO_2_) and segmented data (blue: brine; red: CO_2_), (b) at the end of imbibition, (c) after 58 h of storage, and (d) after injecting one pore volume of brine. (e) CO_2_ saturation profile along the sample. (f) Porosity distribution along the sample. The shaded regions in (e) and (f) represent uncertainties associated with image segmentation.

After 58 h of storage, the trapped CO_2_ saturation remained stable, suggesting that Ostwald ripening has a minimal impact on overall saturation. This diffusion-driven process redistributes mass via curvature-driven solubility gradients while conserving total volume.^14,16,18^ Following the subsequent injection of up to 1 pore volume of brine, the trapped CO_2_ saturation decreased significantly to 15.6%. This reduction is likely due to the rearrangement of the CO_2_ ganglia during storage—i.e., Ostwald ripening that reconnects a fraction of the initially trapped CO_2_. Similar effects of Ostwald ripening in enhancing H_2_ withdrawal have been reported by Goodarzi et al.^14,16^ and Zhang et al.^15^ The reduction in residual saturation is consistent with pore-scale modeling that predicted a 20–25% reduction in residual saturation due to Ostwald ripening:^23^ we see a larger reduction of 32%, which may be caused by some dissolution of CO_2_ during the final brine injection.

### 3.2 Qualitative Characterization of CO_2_ Ganglia Evolution

Figure 3 presents two- and three-dimensional visualizations of the CO_2_ ganglia at different stages. At the end of imbibition (Figures 3a, 3d, 3g, and 3j), the trapped CO_2_ ganglia exhibit a wide range of sizes and morphologies, consistent with observations by Andrew et al.^11^ and Iglauer et al.^32^ During Ostwald ripening, significant changes occur in ganglia size, morphology, and connectivity. Ganglia with high interfacial curvature—particularly small, isolated ones—tend to shrink, while neighboring ganglia with lower curvature expand, often accompanied by coalescence and enhanced overall connectivity (Figures 3b and 3e). However, such expansion and coalescence in the complex pore networks are not unrestricted. As ganglia grow, they invade adjacent throats, increasing local interfacial curvature and triggering localized dissolution at these constrictions, followed by mass transfer to nearby ganglia (Figures 3g and 3h). Additionally, ganglia fragmentation is also observed, likely due to mass-transfer-induced snap-off, which reduces the ganglion volume and weakens connectivity (Figures 3j and 3k). Following 1 pore volume brine injection, the CO_2_ ganglia—initially trapped by capillary forces, then rearranged and reconnected via Ostwald ripening—are ultimately displaced, as shown in Figures 3c, 3f, 3i, and 3l.

**Figure 3:**
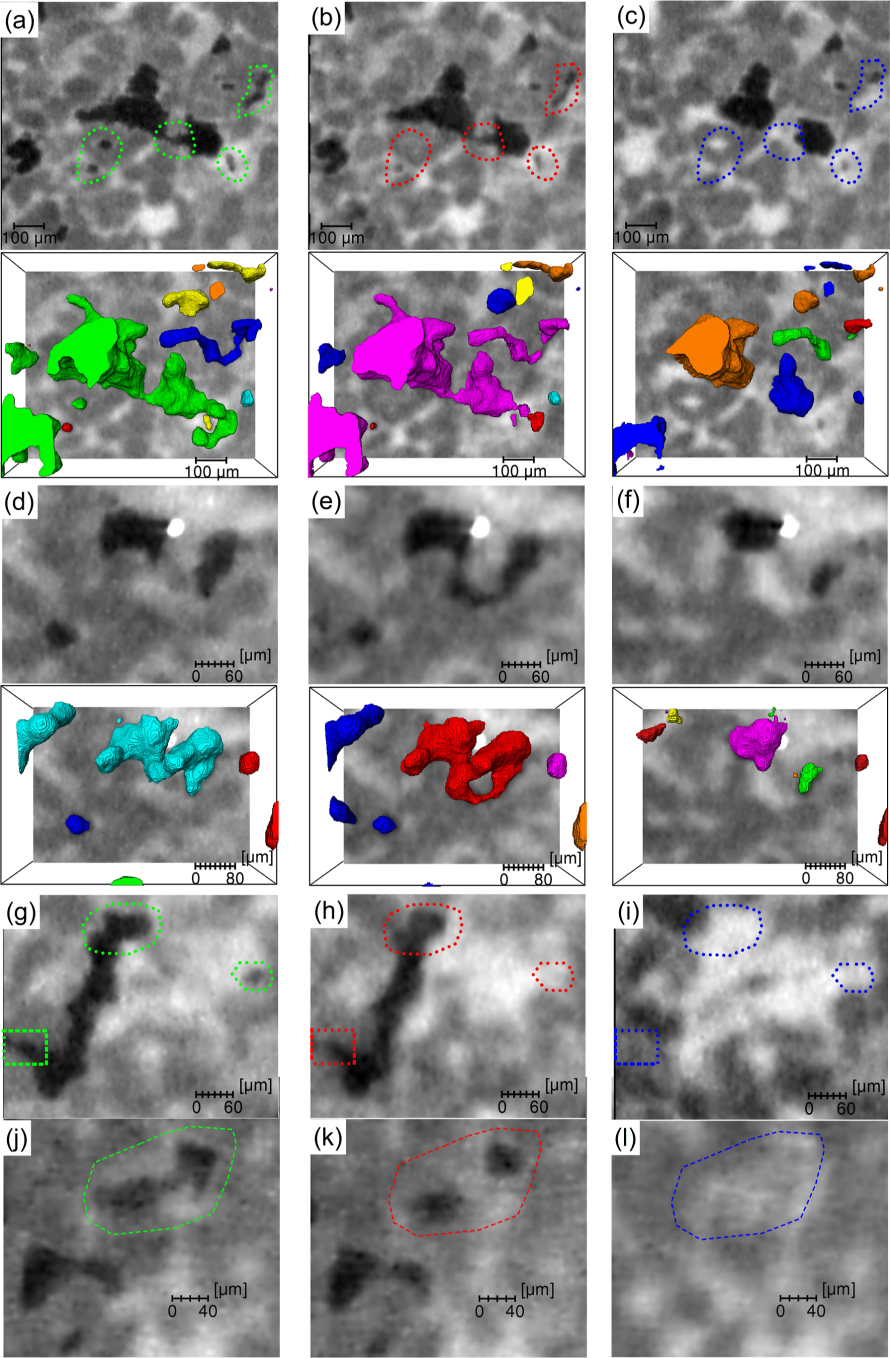
Visualization of CO_2_ ganglia evolution at different stages. Two-dimensional images (black represents CO_2_, gray denotes rock, and white indicates brine) and the corresponding three-dimensional renderings (with individual CO_2_ ganglia shown in distinct colors) acquired at the end of imbibition (a,d,g,j), after 58 h of storage (b,e,h,k), and following the injection of one pore volume of brine (c,f,i,l).

The ganglia rearranged by mass transport of CO_2_ dissolved in the brine.^28,34^ This process aims to make the local capillary pressure constant by promoting the dissolution of high-curvature ganglia and the growth of low-curvature ones.^35^ Higher interfacial curvature elevates CO_2_ chemical potential via increased Gibbs surface excess energy from the Laplace capillary pressure,^36^ thereby enhancing equilibrium solubility and local dissolved CO_2_ concentrations in the brine; lower curvature has the opposite effects. This disparity creates concentration gradients that drive diffusive mass transfer through the aqueous phase from high- to low-curvature regions, ultimately resulting in ganglion shrinkage, expansion, coalescence, and fragmentation as the system evolves toward equilibrium.

### 3.3 Quantification of CO_2_ Ganglia Evolution

Figure 4 quantifies the evolution of the CO_2_ ganglia at different stages. As shown in Figure 4a, at the end of imbibition, ganglia exhibit a broad size distribution, with the largest volume reaching 7.17 × 10^7^ μm^3^. Over 58 h of storage, corresponding to the Ostwald ripening process, the proportion of ganglia with volumes less than 2.2 × 10^3^ μm^3^ and greater than 8.5 × 10^6^ μm^3^ decreases, while the fraction within this intermediate range increases. Specifically, a clear size-dependent behavior is observed during Ostwald ripening in Figures 4b and 4c: small ganglia tend to dissolve and disappear; intermediate ganglia either shrink or grow; and large ganglia remain relatively stable, though occasional fragmentation occurs. Subsequent injection of 1 pore volume of brine mobilizes and displaces the rearranged, large, and connected ganglia, while small isolated ones remain trapped and occupy a growing proportion of residual CO_2_. As shown in Figures 4d and 4e, brine displacement not only induced complete dissolution, growth, shrinkage, and coalescence of CO_2_ ganglia but also significantly enhanced the fragmentation of large ganglia, likely driven by flow-induced snap-off.^37,38^

**Figure 4:**
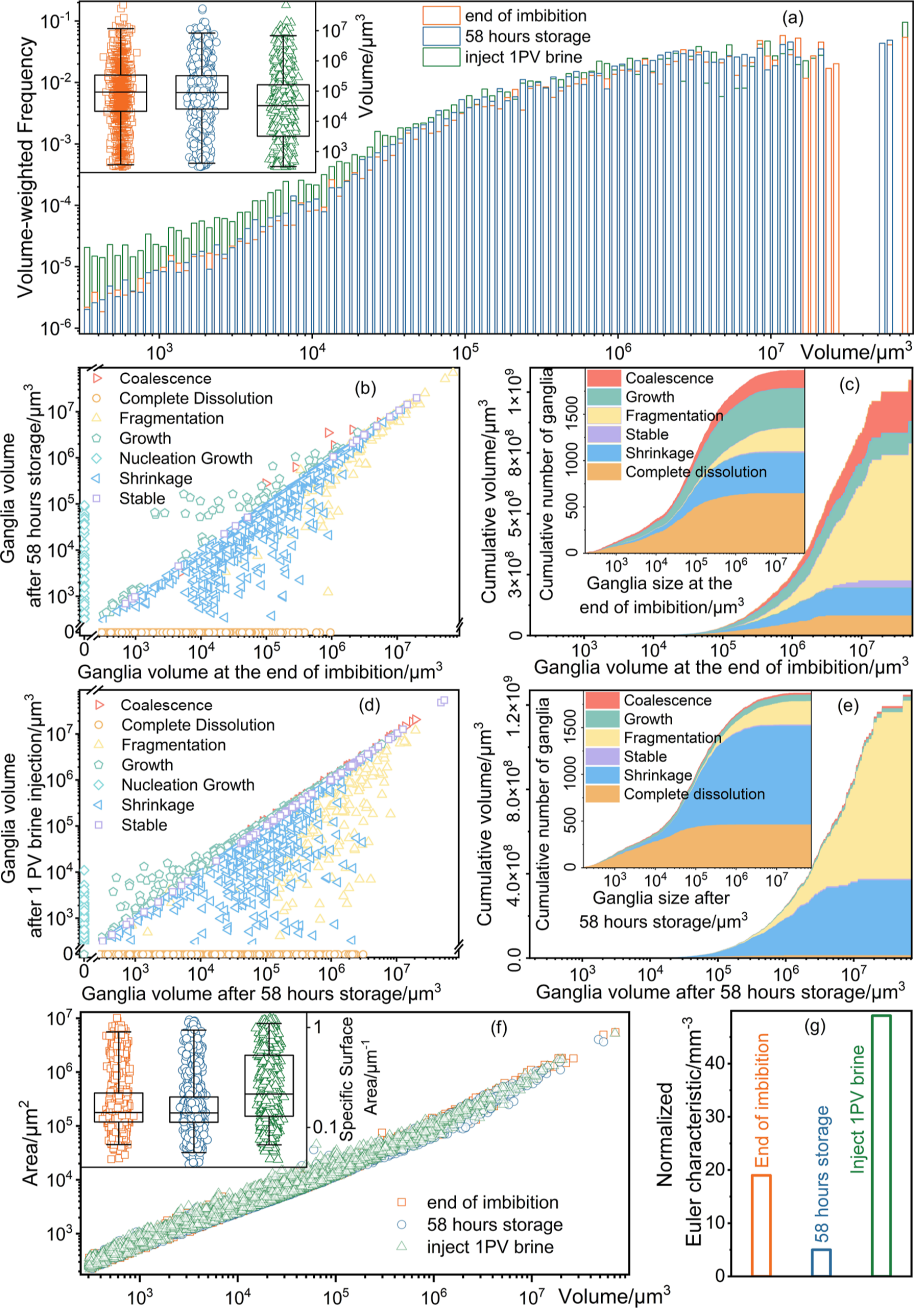
Statistical analysis of CO_2_ ganglia evolution at different stages. (a) Statistical analysis of CO_2_ ganglia volume at the end of imbibition, after 58 h of storage, and following the injection of one pore volume of brine. (b,d) Evolution of ganglion volumes between stages and (c,e) corresponding statistical analyses of cumulative volume and ganglion count relative to the end of imbibition and poststorage, respectively, excluding nucleation growth. (f) Specific surface area of CO_2_ ganglia at different stages. (g) Normalized Euler characteristic^14,16^ of CO_2_ ganglia at different stages.

The specific surface area, defined as the ratio of a ganglion’s surface area to its volume, serves as a quantitative descriptor of ganglion morphology. Lower values correspond to more regular, spherical shapes with reduced morphological complexity.^39^ As shown in Figure 4f, the average specific surface area of ganglia decreases during Ostwald ripening. This trend reflects the progressive elimination of morphologically complex ganglia and a transition toward more spherical, energetically favorable configurations resulting from ganglion coarsening and rearrangement. During subsequent brine injection, ganglia with more regular shapes are preferentially mobilized and displaced. In contrast, those with higher specific surface areas—indicative of irregular or elongated morphologies—are more likely to remain trapped within the pore space.

The Euler characteristic^26,40^ quantifies fluid connectivity in porous media, with lower values indicating enhanced connectivity. As indicated in Figure 4g, initially, the normalized Euler characteristic of the CO_2_ ganglia was measured at 19 mm^–3^, indicating trapped ganglia. Following Ostwald ripening, this value decreased to 5 mm^–3^, signifying better ganglion connectivity over time. This transition is likely driven by the dissolution and disappearance of small, isolated ganglia as well as the coalescence of neighboring ganglia through the transport of dissolved CO_2_, as illustrated in Figure 3. A comparable increase in connectivity has been observed in H_2_ systems undergoing Ostwald ripening.^14–16^ During subsequent brine injection, these interconnected ganglia were preferentially displaced, leaving behind isolated ganglia with a high normalized Euler characteristic of 35 mm^–3^.

Transport of dissolved CO_2_ in the aqueous phase, Ostwald ripening, can drive the rearrangement of capillary-trapped CO_2_ in the pore space and potentially reduce residual saturation. High-resolution 3D X-ray imaging was employed to investigate this in situ process at the pore scale. The experiments demonstrated that the rearrangement of the CO_2_ ganglia during Ostwald ripening reconnects previously trapped CO_2_, reducing its saturation from 22.8% to 15.6%. The morphological evolution of CO_2_ ganglia is driven by simultaneous growth and dissolution, accompanied by reductions in morphological complexity, increases in sphericity, and enhanced connectivity. Ganglion size-dependent behavior is evident: small ganglia dissolve and disappear; intermediate ones exhibit either growth or shrinkage, while large ganglia remain largely stable, with occasional fragmentation. Our study provides the first quantitative analysis of Ostwald ripening’s effect on CO_2_ trapping efficiency, offering in situ insights into the evolution of CO_2_ ganglia. These findings align with those of Xu et al.^18^ and Feng et al.,^19^ which emphasize the critical role of Ostwald ripening in porous media. While our imaging and quantitative analysis captured these Ostwald ripening dynamics, we did not identify a definitive threshold for interganglion spacing that universally triggers or accelerates ripening. This limitation arises from the complexity of natural porous media, where ripening transcends simple distance metrics and is influenced by several interdependent factors: heterogeneous pore structures that create tortuous diffusion pathways, variable capillary barriers that modulate brine connectivity, and diverse ganglion sizes that shape local concentration gradients. Consequently, Ostwald ripening proceeded continuously across the observed separations, with even remotely positioned small ganglia dissolving via viable pathways, underscoring the process’s inherently probabilistic and system-dependent nature rather than a simplistic binary threshold.

While this work was conducted at the scale of a few millimeters, it does have implications for field-scale displacement. Since Ostwald ripening is a diffusive process, reaching equilibrium through transport in the aqueous phase alone at meter scales or larger is extremely slow,^20,35^ taking thousands to many millions of years. However, what our work shows is that over a few days, trapped gas ganglia can reconnect.^14,15^ This means that gas can be transported in its own phase through advective flow, which is potentially much more rapid.^28,41^ Our work suggests that initially trapped gas can begin to flow and that there can be further displacement with field-scale consequences. In large-scale modeling, the end result is simply a reduction in local residual saturation: in our work, we see a reduction of approximately one-third compared to measured values with immiscible fluids or from experiments done quickly so that Ostwald ripening has not had sufficient time to reconnect some of the gas. In addition, there is a need to correct relative permeabilities and capillary pressures measured too rapidly to account for Ostwald ripening to better represent field conditions where equilibrium is likely to be reached, at least over length scales sufficient to affect advective transport. Incorporating Ostwald ripening through longer experimental durations is an option. In addition, the approach to use could follow a simple rescaling of the saturation dependence, using the adjusted lowered residual saturation as suggested by pore-scale modeling.^23^

Building on this research on the Ostwald ripening mechanisms, future research will examine the effects of temperature, pressure, salinity, and heterogeneity on the ripening rates in porous media. Temperature effects may be competing: higher temperatures could enhance molecular diffusion rates and accelerate ripening, yet reduce CO_2_ solubility,^42^ diminishing chemical potential gradients and potentially slowing kinetics, with net outcomes dependent on the exact conditions. Similarly, elevated pressures could enhance CO_2_ solubility via higher partial pressures, amplifying concentration gradients and diffusive fluxes, but could concurrently suppress diffusion rates and retard the process. Salinity gradients, increasing with depth in formations, may reduce CO_2_ solubility via the salting-out effect,^43^ weakening mass transfer gradients and slowing ripening. Heterogeneity might accelerate ripening in high-connectivity zones while slowing it in low-permeability regions, yielding uneven CO_2_ distributions; additionally, capillary entry pressure may hinder interblock mass transfer, and end effects could enhance local trapping at block boundaries, influencing dissolution and growth dynamics. Moreover, in larger-scale heterogeneous media, a likely scenario is that CO_2_ ganglia reconnect via Ostwald ripening, enabling advective flow in their own phase—a process far faster than mass diffusion.

## Supplementary Material



## Notes

Sample orientation and length effects on Ostwald ripening: quantitative analysis of gravitational forces and comparison with existing literature; and determination of experimental time scales: time scale of Ostwald ripening and justification of shut-in time (PDF)
