# Supplementary material for: Pore-Scale Imaging to Quantify the Evolution and Reduction in Trapped CO2 due to Ostwald Ripening
Source: Environ Sci Technol. 2025 Dec 1;59(49):26419–27. doi: 10.1021/acs.est.5c06424 (PMC12713769; doi:10.1021/acs.est.5c06424)
Supplement: Supplementary file 1 [file es5c06424_si_001.pdf]

Supporting Information: Pore-scale Imaging to Quantify the Evolution and Reduction in Trapped CO_2 due to Ostwald Ripening

Rukuan Chai,[*,†,¶] Sajjad Foroughi,[†] Sepideh Goodarzi,[†] Anindityo Patmonoaji,[†] Foo Yoong Yow,[‡] Branko Bijeljic,[†] and Martin J. Blunt[†]

†*Department of Earth Science and Engineering, Imperial College London, London SW7 2AZ, United Kingdom*

‡*Petroliam Nasional Berhad, PETRONAS, Selangor 43000, Malaysia*

¶* *Corresponding author*

E-mail: r.chai@imperial.ac.uk

Table of Contents

Text S1. Sample Orientation and Length Effects on Ostwald Ripening S-3

1 Quantitative Analysis of Gravitational Forces S-3

2 Comparison with Existing Literature S-3

Text S2. Determination of Experimental Time Scales S-5

1 Time Scale of Ostwald Ripening S-5

2 Justification of Shut-in Time S-5

References S-7

Text S1. Sample Orientation and Length Effects on Ostwald Ripening

We have conducted a quantitative analysis of gravitational forces relative to capillary effects, complemented by a comparison with the relevant literature, to determine that gravitational forces are not significant at the pore scale in our experiments.

1 Quantitative Analysis of Gravitational Forces

To assess the balance between gravitational and capillary forces, we calculated the Bond number, defined as

$$Bo = \frac{\Delta\rho g \delta^2}{\sigma}, \tag{1}$$

where δ is the characteristic length scale;[S1] $\Delta\rho$ is the density difference between phases, $920\,\mathrm{kg/m^3}$;[S2] σ is the interfacial tension, $0.035\,\mathrm{N/m}$;[S3] g is gravitational acceleration, $9.81\,\mathrm{m/s^2}$. $Bo \gg 1$ indicates potential gravity-driven segregation, while $Bo \ll 1$ signifies capillary dominance.

- At the pore/ganglia scale ($\delta = 40\,\mu\mathrm{m}$–the average diameter of pores) $Bo = 4.12 \times 10^{-4}$

Since $Bo \ll 1$, capillary forces overwhelmingly dominate, rendering gravitational effects negligible for fluid redistribution or ripening alterations at scales relevant to trapped CO_2 ganglia. This quantitatively supports our conclusion that Ostwald ripening in this study is primarily capillary-governed, with gravity insufficient to induce buoyancy-driven coalescence or vertical migration of ganglia.

- At the sample scale ($\delta = 0.0237\,\mathrm{m}$–the length of the sample) $Bo = 145$

Here, $Bo \gg 1$ suggests gravity could influence bulk segregation in longer samples, but this is less relevant to localized ripening processes, which occur at the pore scale where Bo remains small.

2 Comparison with Existing Literature

Our findings are consistent with established studies on CO_2 trapping and multiphase flow in short cores, which similarly emphasize the dominance of capillary forces at the pore scale.

- Andrew *et al.*[S4] reported that buoyancy forces were negligible in 10–30 mm long samples, preventing gravitational remobilization of CO_2 after residual trapping.

- Herring *et al.*[S5] found that a CO_2–brine–sandstone system was dominated by capillary and viscous forces in a 21 mm long Bentheimer sandstone, with minimal gravity-induced changes in saturation profiles.

These comparisons reinforce that our vertical, short-sample arrangement minimizes gravitational artifacts for studying capillary-driven ripening.

Text S2. Determination of Experimental Time Scales

1 Time Scale of Ostwald Ripening

In this study, Ostwald ripening initiates from the residual CO_2 trapped after imbibition, resulting in the reorganization of CO_2 ganglia while the overall saturation remains stable. Given the need for sufficient time to observe meaningful ripening effects (as illustrated in the shut-in time justification below)—balanced against practical experimental constraints—we focused on a single time interval of 58 hours. In this interval, we obtained evident Ostwald ripening in porous media as expected from the theoretical analysis below.

2 Justification of Shut-in Time

The shut-in period is grounded in theoretical estimates of the timescale for pore-scale equilibrium and Ostwald ripening of CO_2 ganglia, using the characteristic time equation derived from Blunt[S6] used by Zhang *et al.*[S7] and Moghadasi *et al.*,[S8]

$$t = \frac{Lr^2 P}{3DHRT} \tag{2}$$

where L is the length over which ganglion rearrangement is observed in the pore space, taken here as the sample length in the zoomed-in scan (3.64 mm),[S7,S8] r is the mean pore radius of this sandstone 20 µm, P is the pressure 8 MPa, D is the diffusion coefficient of CO_2 in brine $3.0 \times 10^{-9}\,\mathrm{m^2/s}$,[S9] H is the Henry's law constant $1.35 \times 10^{-4}\,\mathrm{mol \cdot m^{-3} \cdot Pa^{-1}}$,[S2] σ is the interfacial tension 0.035 N/m,[S3] R is the universal gas constant $8.314\,\mathrm{m^3 \cdot Pa \cdot K^{-1} \cdot mol^{-1}}$, and T is the temperature 50 °C. This yields an estimated 28 hours for significant ripening, which is comparable to the 17 hours reported in Moghadasi *et al.*[S8] and provides a conservative lower bound under diffusion-limited conditions.

Complementing this, empirical evidence from analogous CO_2 Ostwald ripening studies supports a 24–30 hour minimum for observable effects: AlZaabi *et al.*[S10] observed ripening in similar properties carbonate rocks by 24 hours; Garing *et al.*[S11] noted fluid redistribution in sample in 30 hours; and Moghadasi *et al.*[S8] reported CO_2 ganglia changes in Bentheimer sandstone within the same timeframe.

Consequently, by integrating the theoretical 28-hour minimum with empirical observations of effects by 30 hours, as well as practical considerations in our experimental arrangement (X-ray CT scanning availability and experimental system stability), we selected a 58-hour shut-in period. This duration exceeds the ripening timescale by approximately 2-fold, thereby allowing for more extensive ganglion ripening and reducing the risk of artifacts

such as incomplete diffusion, while optimally balancing precision and feasibility. In contrast, shorter periods (< 24 hours) could underestimate ripening, whereas excessively long ones (> 100 hours) might introduce secondary processes like mineral alteration.

REFERENCES

(S1) Ewing, R. P.; Berkowitz, B. A generalized growth model for simulating initial migration of dense non-aqueous phase liquids. *Water Resour. Res.* **1998**, *34*, 611–622.

(S2) Linstrom, P. J.; Mallard, W. G. NIST Chemistry WebBook, NIST Standard Reference Database Number 69. 2014.

(S3) Bachu, S.; Bennion, D. B. Interfacial tension between CO_2, freshwater, and brine in the range of pressure from (2 to 27) MPa, temperature from (20 to 125) °C, and water salinity from (0 to 334 000) $mg \cdot L^{-1}$. *J. Chem. Eng. Data.* **2009**, *54*, 765–775.

(S4) Andrew, M.; Bijeljic, B.; Blunt, M. J. Pore-scale imaging of trapped supercritical carbon dioxide in sandstones and carbonates. *Int. J. Greenhouse Gas Control* **2014**, *22*, 1–14.

(S5) Herring, A. L.; Andersson, L.; Schlüter, S.; Sheppard, A.; Wildenschild, D. Efficiently engineering pore-scale processes: The role of force dominance and topology during nonwetting phase trapping in porous media. *Adv. Water Resour.* **2015**, *79*, 91–102.

(S6) Blunt, M. J. Ostwald ripening and gravitational equilibrium: Implications for long-term subsurface gas storage. *Phys. Rev. E* **2022**, *106*, 045103.

(S7) Zhang, Y.; Bijeljic, B.; Gao, Y.; Goodarzi, S.; Foroughi, S.; Blunt, M. J. Pore-scale observations of hydrogen trapping and migration in porous rock: Demonstrating the effect of Ostwald ripening. *Geophys. Res. Lett.* **2023**, *50*, e2022GL102383.

(S8) Moghadasi, R.; Foroughi, S.; Goodarzi, S.; Zhang, Y.; Bijeljic, B.; Blunt, M. J.; Niemi, A. Trapping and remobilization during geological CO_2 storage: A pore-scale imaging and modeling study. *Adv. Water Resour.* **2025**, *205*, 105092.

(S9) Cadogan, S. P.; Maitland, G. C.; Trusler, J. P. M. Diffusion coefficients of CO_2 and N_2 in water at temperatures between 298.15 K and 423.15 K at pressures up to 45 MPa. *J. Chem. Eng. Data.* **2014**, *59*, 519–525.

(S10) AlZaabi, A.; Alzahrani, H. M.; Alhosani, A.; Bijeljic, B.; Blunt, M. J. Wettability, pore occupancy, connectivity and Ostwald ripening of nitrogen, carbon dioxide, and hydrogen in carbonate rocks: A comparative study. *Int. J. Hydrogen Energy* **2025**, *135*, 596–608.

(S11) Garing, C.; Benson, S. M. Pore-Scale imaging of multiphase flow in porous media: Towards improved predictions of relative permeability and capillary pressure. American Geophysical Union Fall Meeting. San Francisco, CA, 2019; Abstract H41E-05.
